# Spirometrology

**Published:** 1878-06

**Authors:** 


### HALL'S JOURNAL OF HEALTH.

Our Legitimate Scope is almost boundless: for whatever begets pleasurable and harmless feelings, promotes Health; and whatever induces disagreeable sensations, engenders Disease.

WE AIM TO SHOW HOW DISEASE MAY BE AVOIDED, AND THAT IT IS BEST, WHEN SICKNESS COMES, TO TAKE NO MEDICINE WITHOUT CONSULTING A PHYSICIAN

Vol. 25 JUNE, 1878 [No. 6.

### SPI-RO-ME-*TROL*-O-*GY*,

PRONOUNCED with the accent on the ante-penult, orfourth syllable, teaches the measurement of the breath, and, by a little license, the lungs themselves, as the breath is contained in the lungs. If a man has all his lungs within him, in full operation, it is impossible for him to have consumption, whatever may be his symptoms, because consumption is a destruction of a portion of the lungs, and when that is the case they can no more have the full amount of breath or air than a gallon measure can hold a gallon after its size has been diminished by having a portion of the top off or removed.

It becomes, then, of great importance to accomplish two things:—

First, to measure accurately, and with as much certainty as you would measure wheat by a standard and authentic bushel measure, the amount of air contained in the lungs.

Second, to ascertain what amount of air the lungs ought to contain in full and perfect health.

The chemist has no difficulty in measuring out to you a cubic foot of gas. The gas which lights our dwellings and which burns in the streets of cities, when the moon don't shine, is *capable* of being accurately measured, and so is the air we breathe, with equal simplicity and certainty, even to the fraction of a cubic inch.

That the amount thus contained is proportioned to the man's height.

That that proportion is eight cubic inches of air for every additional inch of height above a certain standard.

With these facts, now admitted as such, inferences may be drawn of great interest in connection with other observations, which any reader who takes the trouble may verify.

Observation 1st.—I have never known a man who was in admitted consumption, and whose subsequent death and *post-mortem* confirmed the fact, capable of measuring his full standard.

Observation 2d.—In numerously repeated instances, persons have been pronounced to have undisputed consumption, and as such were abandoned to die, but on measurement they have reached their full standard, enabling me to say that they had not consumption, and their return to good health, and their continuance in it for years after, and to this day, is an abiding proof of the correctness of my decision.

Observation 3d.—No persons have come under my care, who died of consumption within a year, who, at the time of examination reached their full lung measurement.

Observation 4th.—Therefore, any man who reaches his standard, has reason to believe that he cannot die of consumption within a year, an assurance which, in many cases, may be of exceeding value.

Observation 5th.—As a man with healthy lungs always reaches his full standard, and as it is impossible for a consumptive man to measure his full standard, then it may be safely concluded that a man cannot die of consumption while he gives his healthy measure, and also that he who cannot measure full, is in danger, and should not rest a single day, until he can measure to the full.

When persons are under medical treatment for deficient lung measurement, accompanied with the ordinary symptoms of common consumption, they improve from week to week in proportion as they measure out more and more air from the lungs: on the other hand, when they measure less and less from time to time, they inevitably die. With this view of the case, the reader will perceive that as a general rule a man can tell for himself, as well as his physician, whether he is getting well or not, and, as an illustration, an article is copied verbatim from the eighth edition of "Bronchitis and Kindred Diseases," page 361, on

